# 

**Published:** 1897-10

**Authors:** 


### BOOKS RECEIVED.

Tuberculosis of the Genito-Urinary Organs, Male and Female. By N. Senn, M. D., LL. D., Professor of Practice of Surgery and Clinical Surgery, Rush Medical College; Attending Surgeon to Presbyterian Hospital; Surgeon-in-Chief, St. Joseph's Hospital, Chicago. Octavo, pp. vi.—317. Illustrated. Price, $3.00, net. Philadelphia: W. B. Saunders, 925 Walnut street. 1897.

The American Academy of Railway Surgeons. Report of the third annual meeting, held at Chicago, Ill., September 23, 24 and 25, 1896. Edited by R. Harvey Reed, M. D., Columbus, O. Chicago: American Medical Association Press. 1897.

Report of the Commissioner of Education for the Year 1895-96. Volume I. Containing Part I. Washington: Government Printing Office. 1897.

Transactions of the American Gynecological Society. Volume XXII. For the Year 1897. J. Riddle Goffe, M. D., Secretary. Philadelphia: Wm. J. Dornan, Printer. 1897.

The Origin of Disease, Especially of Disease Resulting from Intrinsic as Opposed to Extrinsic Causes. With chapters on diagnosis, prognosis and treatment. By Arthur V. Meigs, M. D., Physician to the Pennsylvania Hospital. Octavo, pp. xiv.—229. With 137 original illustrations. Philadelphia: J. B. Lippincott Co. 1897.

The Normal and Pathological Circulation in the Central Nervous System (Myel-Encephalon). Original Studies by William Browning, Ph. B., M. D., Attending Neurologist to the Kings County Hospital and Consulting to the St. Christopher's Hospital for Babies, etc., etc. Small 8vo, pp. 171. Price, $1.50. Philadelphia: J. B. Lippincott Co. 1897.

Transactions of the Medical Society of the State of West Virginia, held at Charleston, May 19, 20 and 21, 1897. Instituted April 10, 1867.

Principles of Medicine. Designed for use as a text-book in medical colleges and for consideration by practitioners generally. By Charles S. Mack, M. D., Professor of Materia Medica and Therapeutics in the Hahnemann Medical College and Hospital, Chicago. Price, $1.00. Chicago: The W. T. Keener Co. 1897.

Atlas and Essentials of Bacteriology. By Professor K. B. Lehmann, Würzburg, and Dr. Rudolf Neumann, Würzburg. Sixty-three chromolithographic plates, comprising 558 separate figures, with explanatory text facing plates, and 128 page treatise, illustrated. Muslin, $3.00, net. New York: William Wood & Co. 1897.

